# Personal

**Published:** 1893-08

**Authors:** 


### Personal.

DR. FRANK W. ABBOTT has removed his office and residence from 223 to 523 Franklin street, between Allen and North streets. Office hours, 9 A. M. to 1 P. M. Other hours by appointment. Telephone, 1930.

---

DR. JOHN PARMENTER, Professor of Anatomy and Adjunct Professor of Clinical Surgery in the Medical Department of the University of Buffalo, was elected Fellow of the American Surgical Association at its recent meeting held in Buffalo. This is a deserved recognition of the surgical talent of one of the younger members of the profession.

---

DR. J. D. FLAGG has been elected Professor of Physiology in Niagara University, in the place of Dr. Geo. E. Fell, resigned; Dr. Harry A. Wood has been appointed Adjunct Professor of Materia

Medica and Therapeutics; Dr. L. Bradley Dorr has been made Lecturer on Bacteriology, in addition to his Adjunct Professorship of Chemistry; Dr. David L. Redmond has been elected Lecturer on Dermatology; Dr. Frederick A. Hayes, Demonstrator of Anatomy; Dr. Edward M. Dooley, Lecturer on Anatomy; Dr. Frederick Preiss, Assistant to the Chair of Principles and Practice of Surgery; and Dr. Wm. G. Taylor, Clinical Assistant in Obstetrics.

---

Dr. John B. Hamilton, Professor of Surgery in Rush Medical College, Chicago, Ill., has been appointed editor of the *Journal of the American Medical Association.* Dr. Hamilton's experience as a man of affairs, his wide professional acquaintance, and his knowledge of journalism, will increase the usefulness and prosperity of the *Journal* in a marked degree. It requires no argument to prove that this periodical has not been in the past, under any administration, what it deserved to be. Let us hope that Dr. Hamilton's advent auspiciously inaugurates an era of prosperity and influence for the *Journal* which ought to represent the profession of America.

---

Among the Buffalo physicians who attended the Medical Association of Central New York, held June 16th, at Rochester, were, Drs. Bartlett, Ingraham, Hubbell, Congdon, Howe, Twohey, Thornbury, Benedict, Crego, Preiss, and Krauss. Dr. H. L. Elsner, of Syracuse, was elected President; Dr. F. S. Crego, of Buffalo, was elected Vice-President of the Association for the ensuing year. The next meeting will take place in Buffalo.

---

Messrs. Hummel & Parmele, the well-known medical journal advertising agents, have removed from the Drexel building to more commodious quarters, at No. 257 South Fourth street, Philadelphia, Pa.

---

At the June commencement, Niagara University conferred the degree of LL. D. upon Dr. John Cronyn, and the degree of Ph. D. upon Drs. Thomas Lothrop and Alvin A. Hubbell, of Buffalo, and Dr. Frederick Peterson, of New York.

---

Dr. Maud J. Frye, late of the Woman's Hospital, Detroit, has located at 224 Allen street, Buffalo, N. Y.

